# Hepatitis E Virus Genotypes and Evolution: Emergence of Camel Hepatitis E Variants

**DOI:** 10.3390/ijms18040869

**Published:** 2017-04-20

**Authors:** Siddharth Sridhar, Jade L. L. Teng, Tsz-Ho Chiu, Susanna K. P. Lau, Patrick C. Y. Woo

**Affiliations:** 1 Department of Microbiology, Li Ka Shing Faculty of Medicine, The University of Hong Kong, Hong Kong, China; sid8998@gmail.com (S.S.); llteng@hku.hk (J.L.L.T.); tsz.chiu@uqconnect.edu.au (T.-H.C.); skplau@hku.hk (S.K.P.L.); 2 State Key Laboratory of Emerging Infectious Diseases, Department of Microbiology, Li Ka Shing Faculty of Medicine, The University of Hong Kong, University Pathology Building, Queen Mary Hospital, Hong Kong, China; 3 Research Centre of Infection and Immunology, Li Ka Shing Faculty of Medicine, The University of Hong Kong, Hong Kong, China; 4 Carol Yu Centre for Infection, Li Ka Shing Faculty of Medicine, The University of Hong Kong, Hong Kong, China

**Keywords:** hepatitis E, zoonosis, hepatitis, taxonomy, molecular evolution, molecular epidemiology, genotypes, dromedary camel, Bactrian camel, swine

## Abstract

Hepatitis E virus (HEV) is a major cause of viral hepatitis globally. Zoonotic HEV is an important cause of chronic hepatitis in immunocompromised patients. The rapid identification of novel HEV variants and accumulating sequence information has prompted significant changes in taxonomy of the family Hepeviridae. This family includes two genera: *Orthohepevirus*, which infects terrestrial vertebrates, and *Piscihepevirus*, which infects fish. Within *Orthohepevirus*, there are four species, A–D, with widely differing host range. *Orthohepevirus* A contains the HEV variants infecting humans and its significance continues to expand with new clinical information. We now recognize eight genotypes within *Orthohepevirus* A: HEV1 and HEV2, restricted to humans; HEV3, which circulates among humans, swine, rabbits, deer and mongooses; HEV4, which circulates between humans and swine; HEV5 and HEV6, which are found in wild boars; and HEV7 and HEV8, which were recently identified in dromedary and Bactrian camels, respectively. HEV7 is an example of a novel genotype that was found to have significance to human health shortly after discovery. In this review, we summarize recent developments in HEV molecular taxonomy, epidemiology and evolution and describe the discovery of novel camel HEV genotypes as an illustrative example of the changes in this field.

## 1. Introduction

Hepatitis E virus (HEV) is an important cause of both epidemic and sporadic viral hepatitis. HEV has been estimated to cause 3.3 million symptomatic cases of acute hepatitis worldwide [1] and in some areas has overtaken hepatitis A as the most common cause of acute viral hepatitis [2]. Although most cases are self-limiting, pregnant women, in particular, have a propensity to progress to fulminant hepatitis [3]. Furthermore, immunocompromised patients such as transplant recipients, patients with hematological malignancies and advanced HIV may progress to chronic hepatitis following an HEV infection [4,5,6]. 

The global distribution of human disease due to HEV relies partly on its “two-faced” epidemiology. In hyperendemic areas, hepatitis E is often linked to contamination of water supplies with human fecal material and represents person-to-person transmission [7]. However, in low endemic areas with high socioeconomic development, hepatitis E is predominantly a foodborne zoonosis, which is most commonly transmitted to humans consuming undercooked pork products [7,8]. In addition to pork, hepatitis E has also been transmitted to humans consuming meat or milk of feral animals including wild boar, deer, rabbits and camels [9,10,11,12,13]. 

The basis for this contrasting epidemiology lies in the phylogenetic diversity of HEV. Our knowledge of HEV diversity is constantly evolving with the discovery of novel HEV variants in various animal hosts around the world. With the accumulation of high-quality genomic information, HEV taxonomy has undergone significant changes recently to reflect the diversity of this family of viruses. Sophisticated sequence analyses have been possible to address various questions related to the molecular epidemiology and evolution of HEV. The objective of this review is to provide an update on the molecular basis of modern HEV taxonomy along with a summary of recent advances in HEV epidemiology and evolution. The discovery of camelid HEV genotypes and recognition of their importance to human health is used as an illustrative example of recent changes in this field.

## 2. HEV Genomic Organization

**Figure 1:**
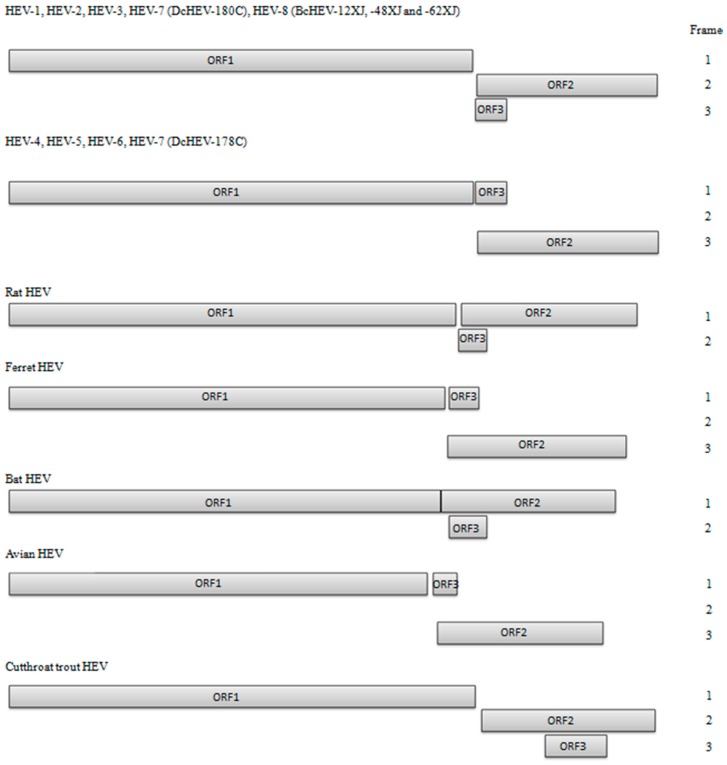
Predicted genomic organization of HEV-1 to HEV-8, considering the reading frame of ORF1 as frame 1. Reading frames of ORF2 and ORF3 are labeled relative to ORF1 reading frame. HEV-1 (M73218); HEV-2 (M74506); HEV-3 (AP003430); HEV-4 (AJ272108); HEV-5 (AB573435); HEV-6 (AB602441); HEV-7 strain DcHEV-178C (KJ496143); HEV-7 strain DcHEV-180C (KJ496144), HEV-8 (KX387865).

HEV is a nonenveloped virus with a 27–34 nm, *T* = 1 icosahedral capsid enclosing the viral genome. The single-stranded, positive-sense RNA genome varies from 6.6 to 7.3 kB in length; similar to eukaryotic mRNA, the 5′ end has a 7-methylguanylate (m^7^G) cap while the 3′ end is polyadenylated [14]. Short untranslated regions flank three open reading frames (ORFs), which are named ORF1, ORF2 and ORF3 in a 5′→3′ direction. Overlapping reading frames ORF2 and ORF3 encode for frameshifted products from a bicistronic subgenomic mRNA species [15]. There is inter-species variation in the location of the ORF2/ORF3 junction with the divergent variant cutthroat trout virus having an ORF3 that is displaced towards the middle of ORF2 (Figure 1).

ORF1 is the longest of the three reading frames, accounting for nearly two-thirds of the entire genome [14]. It encodes a non-structural polyprotein that possesses several functional domains critical for viral replication [16]. There is still controversy regarding whether the ORF1 product undergoes any post-translational proteolytic processing [17]. Functionally characterized domains of ORF1 include a methyltransferase, helicase and RNA-dependent RNA polymerase (RdRp). Computational analysis of ORF1 also reveals a papain-like cysteine protease, macro and hypervariable domains. The exact role of these domains in the HEV replication cycle remains speculative and may indirectly be inferred by homologous domains in other positive-sense RNA viruses [16].

The ORF2 region on the 2.2 kB m^7^G capped subgenomic RNA (sgRNA) species encodes the viral capsid protein, which is important for viral entry and also contains immunological epitopes for antibody targets [17]. The ORF3 region is also expressed from the same sgRNA species and the start codons of ORF2 and ORF3 are only separated by only a few nucleotides [14]. The ORF3 encoded phosphoprotein has been observed to interact with the cellular cytoskeleton and endosomal system and is tentatively believed to play a role in viral egress [18,19].

## 3. Hepatitis E Virus (HEV) Taxonomy: In Constant Evolution

HEV is difficult to culture from clinical specimens, animal models are not widely available and several HEV variants from diverse epidemiological sources appear to have broadly cross-reactive antigenicity [20,21,22,23]. Therefore, from an early stage, the genetic characteristics of HEV variants were the main basis for taxonomic classification as opposed to other viral families where serotyping and cell culture characteristics played an important role in early classification schema. Classifications of HEV using genetic methods also closely mirror ecology and zooepidemiology of virus variants proving the fundamental soundness of this technique. However, as more sequence information from divergent isolates became available, there was considerable confusion due to the proliferation of differing classification schemes based on methods such as nucleotide identities, genetic distances, amino acid identities, phylogenetic analysis, PCR-restriction fragment length polymorphism, and ORF structure of different areas of the genome [24,25,26,27,28,29,30,31]. Indeed, the very definition of various taxons within Hepeviridae has undergone continuous evolution to reflect the emerging diversity of the family.

Originally included as a member of the family Caliciviridae, HEV was reassigned to the newly-created family Hepeviridae in 2009 by the International Committee on Taxonomy of Viruses (ICTV). According to the current taxonomy release (2015), the family Hepeviridae includes two genera: *Orthohepevirus* and *Piscihepevirus* [32]. The former encompasses all mammalian and avian HEV variants while the latter includes the highly divergent cutthroat trout virus placed within a single species. The *Orthohepevirus* genus is further subdivided into four species (A–D). The current consensus proposals for classification of the family Hepeviridae adopted by ICTV were put forward by Smith et al. in 2014 [27,32].

Division of the family into the two aforementioned genera was made straightforward by the clear genetic differences of *Piscihepevirus* A, the sole species within the *Piscihepevirus* genus, from other HEV belonging to the *Orthohepevirus* genus: ORF3 of cutthroat trout virus (the only virus within the *Piscihepevirus* genus so far) is displaced far towards the middle of ORF2 (Figure 1) compared to members of the *Orthohepevirus* genus, and comparative inter-genus *p*-distances of even functionally significant (i.e., highly conserved) amino acid sequences encoded by ORF1 like methyltrasferase, helicase and polymerase exceed 0.55 [27,33]. Furthermore, overall sequence identities of ORF2 and ORF3 between cutthroat trout virus and other members of the Hepeviridae family are only 18–21% and 13‒16%, respectively. In comparison, amino acid sequence similarities of ORF2 and ORF3 between avian and mammalian HEV in the *Orthohepevirus* genus are 42‒55% and 20‒29%, respectively [27]. Table 1 also illustrates other differences in genomic organization between cutthroat trout virus and other HEV types with reference to the newly discovered HEV8 camelid genotype. This extreme phylogenetic divergence is reflected in the ecology of *Piscihepevirus*, which infects fish, in contrast to other members of the family Hepeviridae, which infect terrestrial vertebrates.

## 4. Beyond the Species Level: HEV Genotypes and Subgenotypes

Classification below the species level is beyond the aegis of ICTV; however, guidance on further classification into genotypes have also been proposed by Smith et al. [27]. Much of these classification efforts have focused on *Orthohepevirus* A, which is the most diverse species and contains several genotypes pathogenic to humans. Based on pairwise amino acid sequence comparisons of available concatenated ORF1 and ORF2 sequences (excluding the ORF1 hypervariable region) of HEV variants visualized using a *p*-distance frequency distribution histogram, a *p*-distance of 0.088 appears to mark the demarcation between intra-genotypic and inter-genotypic sequence diversity [27]. A *p*-distance greater than this threshold is, therefore, used as the definition of novel genotypes and this system enables classification of *Orthohepevirus* A into eight genotypes. However, the significant sequence diversity within this species appears to be a continuum and the future discovery of variants that challenge fixed definitions of genotype can be anticipated. The identification of inter-genotypic HEV recombinants infecting swine [31,34] further complicates precise genotype allocations of all strains.

**Figure 2:**
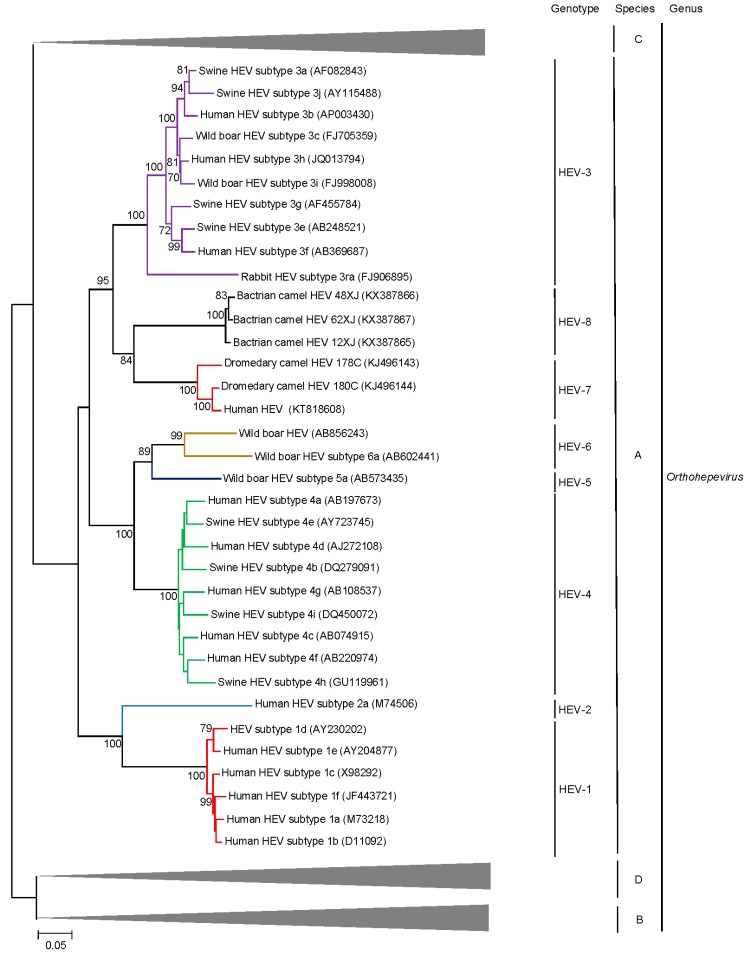
Phylogenetic analyses of ORF1/ORF2 proteins excluding the hypervariable region (HVR) of HEVs within the genus *Orthohepevirus* (Species A to D). The tree was constructed using maximum likelihood method and the optimal substitution model of JTT + G + I was used. In total, 2282 acid positions (amino acid residues 1–706 and 789–2409, numbered with reference to GenBank sequence M73218) were included in the analyses. The scale bar indicates the estimated number of substitutions per 20 amino acids. GenBank accession numbers are shown in brackets.

Individual genotypes within *Orthohepevirus* A are often congruent with well-defined geographical distribution and host restriction although inter-species transmission of particular HEV genotypes has been well documented in both laboratory and field settings [35]. Genotypes 1–4 are most commonly implicated in human infection with contrasting epidemiology. Genotype 1 HEV (HEV-1) only infects humans and is the most common cause of hepatitis E in regions with lower socioeconomic development where it is predominantly spread via contaminated potable water. Genotype 2 HEV (HEV-2) appears to be very rare, the only complete genome sequence comes from a strain identified in Mexico; based on short fragments, some HEV variants identified in Africa may belong to HEV-2 as well [36,37]. HEV-3 and HEV-4 circulate in swine populations (including pigs and wild boar) and are an important cause of zoonotic foodborne autochthonous human hepatitis E in areas with high socioeconomic development [38]. HEV-3 is enzootic in the Americas, Europe and parts of Asia while HEV-4 is enzootic in China and Korea and is emerging as the major cause of human hepatitis E in the region, outcompeting HEV1 in the last decade [39,40,41]. HEV-5 and HEV-6 are novel genotypes identified in Japanese wild boars [42,43] and cluster together with HEV-4 isolates on phylogenetic analysis (Figure 2). HEV-7 and HEV-8 were identified in camels [44,45]; their discovery and characteristics are detailed in the next section as an illustrative example of increasing recognition of Hepeviridae diversity. 

Classification of the other *Orthohepevirus* species into genotypes has also been possible, but intra-species diversity is more limited compared to *Orthohepevirus* A requiring varying definitions of what constitutes a genotype. For example, the divergence among complete genome amino acid sequences of avian HEV (*Orthohepevirus* B) is less than 6%, which is even lower than intra-genotypic diversity in swine HEV-3 [27]. There is sufficient diversity between rodent and ferret HEV to classify *Orthohepevirus* C into two genotypes while there is insufficient sequence information at this stage to further classify bat HEV (*Orthohepevirus* D) [27].

For classification of individual HEV genotypes into subtypes, the initial work of Lu et al. [24] has been expanded further by analysis of nucleotide *p*-distances of all available complete HEV genome sequences and assignment of reference sequences for each subtype [46]. HEV-1 was identified to have six subtypes divisible into two clades, HEV-3 has ten subtypes after excluding the related rabbit HEV clade, and HEV-4 has nine subtypes [46]. Subtype assignment is complex as pairwise *p*-distance variation is often a continuum within and between subtypes; this again precludes setting any fixed demarcation definition of what constitutes a subtype. Several HEV variants still await definitive subtype assignment based on the extant framework. It may be noted that viral subtype has not been correlated with clinical severity or increased inter-species transmissibility [47]. Furthermore, the study of HEV epidemiology, transmission patterns and evolution rely more on identification of clades from sequence datasets rather than precise subtype designations of individual sequences [48,49]. However, the importance of a well curated and labeled database of HEV complete genome or subgenomic sequences as an aid to epidemiological research cannot be overemphasized.

## 5. Discovery of Camel HEV Genotypes

The recent discovery of two camelid HEV genotypes by our group is an illustrative example of our rapidly expanding understanding of HEV ecology [44,45]. In 2014, we reported the discovery of camelid HEV in dromedary camels (*Camelus dromedarius* or one-humped camels) from Dubai and then, in 2016, in Bactrian camels (*Camelus bactrianus* or two-humped camels) from Xinjiang, further expanding the mammalian host diversity of HEV. These camel HEV genomes possessed >20% nucleotide difference to all other HEVs with complete genome sequences available and, therefore, have been proposed as new genotypes of HEV (Table 1): HEV-7 for dromedary camel HEV (DcHEV) and HEV-8 for Bactrian camel HEV (BcHEV). A recent reported case of chronic HEV-7 infection in a liver transplant recipient who regularly consumed camel products [10] suggests the potential high impact to human health of novel genotypes within *Orthohepevirus* A.

**Figure 3:**
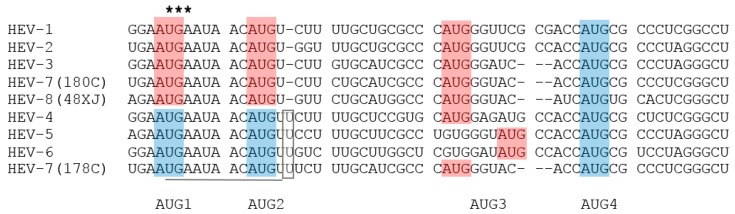
Alignment of nucleotide sequences showing potential start codons for ORF2 and ORF3 in HEV-1 to HEV-8. Potential start codons of ORF3 are shaded by red boxes, and those of ORF2 by blue boxes. The inserted U residues are indicated by open box. The stop codon of ORF1 is marked with asterisks. The conserved *cis*-reactive element with the sequence UGAAUAACAUGU is underlined, which might serve as promoter for the synthesis of the subgenomic mRNA for the ORF2 and ORF3.

Complete genome sequencing was performed on two DcHEV strains (DcHEV 178C and 180C) and three BcHEV strains (DcHEV 12XJ, 48XJ and 62XJ). The genome size of camel HEVs ranged from 7212 to 7223 bases, with G + C content of 52.7% to 55.1% (Table 2). The camel HEV genomes contained three major ORFs, with typical genome organization and characteristics similar to other HEV genotypes of *Orthohepevirus* A (Figure 1). ORF1 polyprotein contained domains consistent with a methyltransferase, a peptide containing a Y-domain, a papain-like cysteine protease, a peptide with a hypervariable region (HVR), a helicase, and an RNA-dependent RNA polymerase. Conserved sequences TLYTRTWS and RRLLXXYPDG that bound the HVR of HEV-1 to HEV-6 [42,50], but not in HEVs of other *Orthohepevirus* species, were also present in camel HEVs. A conserved motif (T/V)SGFSS(X)F(X)P immediately preceding the HVR of HEV-3 to HEV-6, but not present in HEV-1, HEV-2 or HEVs of other *Orthohepevirus* species, were also present in HEV-7 and HEV-8 as VSGFSSDFAP and TSGFSSNFSP respectively. The relative excess of proline and serine observed in the HVR of all other HEVs was also observed for camel HEVs. For DcHEV strain 178C (HEV-7), ORF2 began at nt 5172, similar to HEV-4 to HEV-6 with an insertion of a single nucleotide (U) at nt 5146, and ended at nt 7154, encoding a capsid protein of 660 aa (Table 2; Figure 3). As for DcHEV strain 180C (HEV-7) and the three BcHEV strains 12XJ, 48XJ and 62XJ (HEV-8), because of the lack of U insertion as in HEV-1, HEV-2 and HEV-3, ORF2 began at nt 5171 for DcHEV strain 180C and nt 5176 for the three BcHEV strains, also encoding a capsid protein of 660 aa. For DcHEV strain 178C (HEV-7), similar to HEV-4 to HEV-6, ORF3 began at nt 5161 and ended at nt 5502, encoding a small phosphoprotein of 113 aa with a multifunctional C-terminal region (Table 2; Figure 3). As for DcHEV strain 180C (HEV-7) and the three BcHEV strains (HEV-8), due to the lack of the U insertion as in HEV1, HEV2 and HEV3, ORF3 begain at nt 5160 for DcHEV strain 180C and nt 5165 for the three BcHEV strains (HEV-8) (Table 2; Figure 3). The conserved *cis*-reactive element (UGAAUAACAUGU) located upstream of ORF2 and ORF3 in the camel HEV strains might serve as promoter for the synthesis of the subgenomic mRNA for these two ORFs (Figure 3). Most interestingly, the presence of a U insertion downstream to AUG2 in DcHEV strain 178C resembled those of HEV-4 to HEV-6, leading to three possible start codons (AUG1, AUG2 or AUG4) for its ORF2 but one possible start codon for its ORF3 (AUG3), whereas the lack of this U insertion downstream to AUG2 in DcHEV strain 180C resembled those of HEV1, HEV2 and HEV3 and the three BcHEV strains (HEV-8), leading to only one possible start codon for its ORF2 (AUG4) but three possible start codons for its ORF3 (AUG1, AUG2 or AUG3) (Figure 3). The presence/absence of such a U insertion in different strains of the same HEV genotype has never been observed in other HEV genotypes and is so far unique to DcHEV (HEV-7).

**Figure 4:**
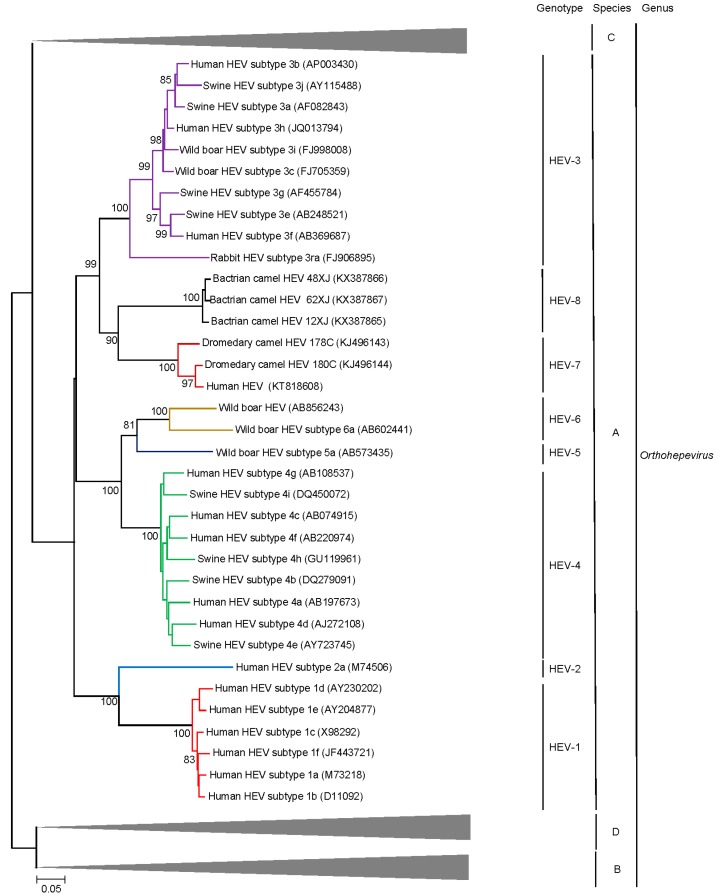
Phylogenetic analyses of ORF1of HEVs within the genus Orthohepevirus (Species A to D). The tree was constructed using maximum likelihood method and the optimal substitution model of JTT + G + I + F was used. Amino acid residues 1–1743 in ORF1, numbered with reference to GenBank sequence M73218, were included in the analyses. The scale bar indicates the estimated number of substitutions per 20 amino acids. GenBank accession numbers are shown in brackets.

**Figure 5:**
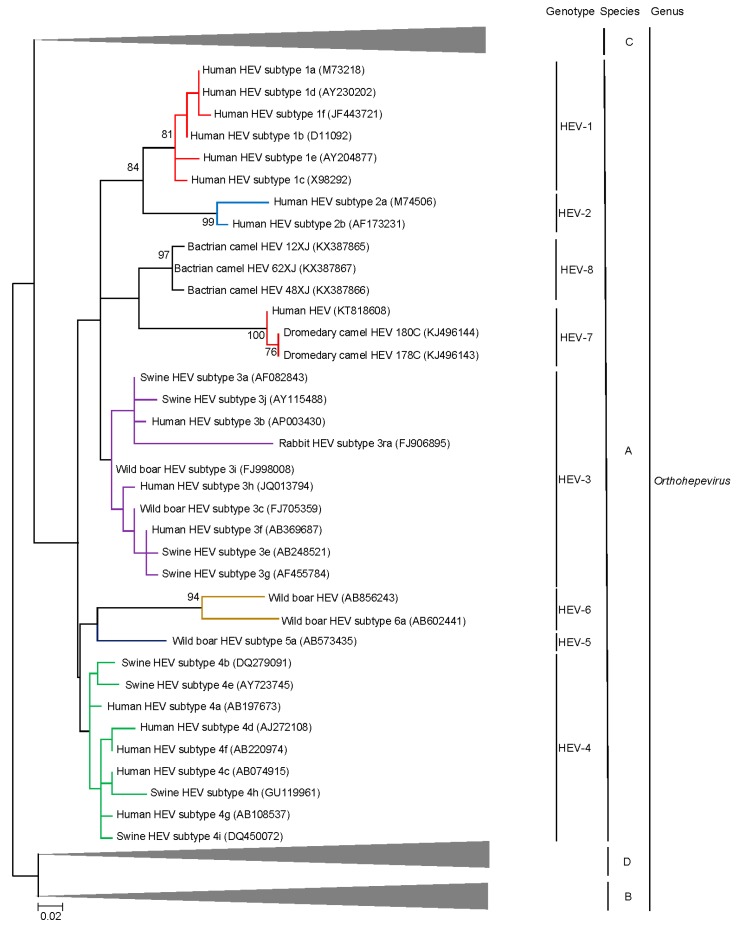
Phylogenetic analyses of ORF2 of HEVs within the genus Orthohepevirus (Species A to D). The tree was constructed using maximum likelihood method and the optimal substitution model of JTT + G + I was used. Amino acid residues 1–660 in ORF2, numbered with reference to GenBank sequence M73218, were included in the analyses. The scale bar indicates the estimated number of substitutions per 50 amino acids. GenBank accession numbers are shown in brackets.

**Figure 6:**
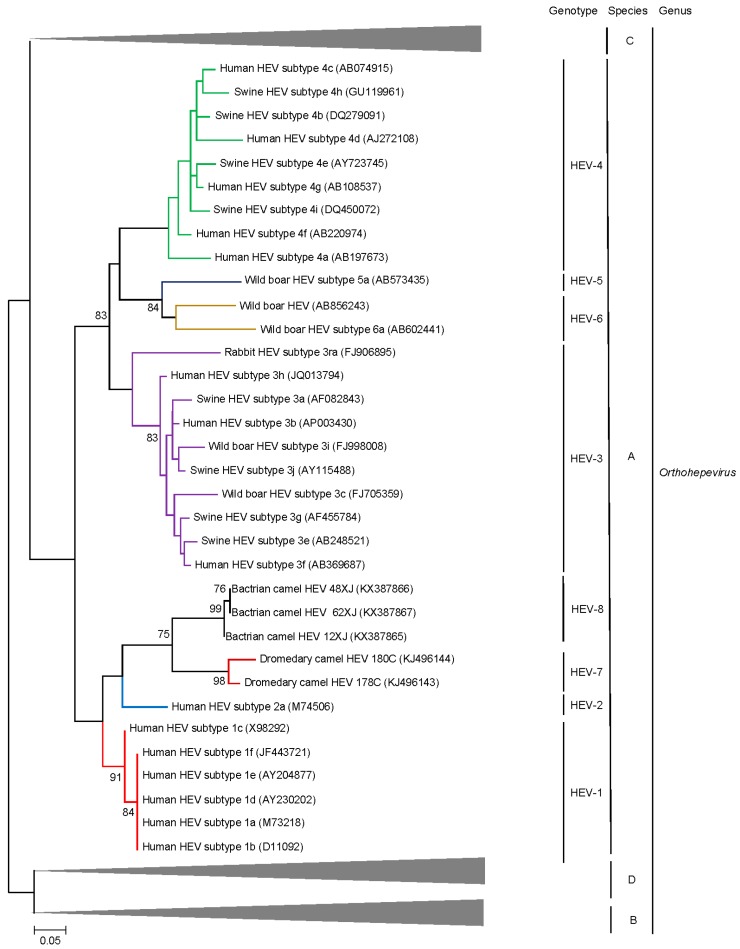
Phylogenetic analyses of ORF3 of HEVs within the genus *Orthohepevirus* (Species A to D). The tree was constructed using maximum likelihood method and the optimal substitution model of JTT + G was used. Amino acid residues 10–123 in ORF3, numbered with reference to GenBank sequence M73218, were included in the analyses. The scale bar indicates the estimated number of substitutions per 20 amino acids. GenBank accession numbers are shown in brackets.

Phylogenetic trees constructed using concatenated ORF1/ORF2 excluding the HVR (Figure 2), ORF1 (Figure 4), ORF2 (Figure 5), ORF3 (Figure 6) and showed that the three Bactrian camel HEV strains (HEV-8) were clustered with the two dromedary camel HEV strains (HEV-7) and the HEV-7 strain from the liver-transplant recipient with chronic hepatitis [10], and they were clustered with different HEVs in different phylogenetic trees. For ORF1 and concatenated ORF1/ORF2 excluding the HVR, HEV-7 and HEV-8 were clustered with HEV-3. However, they were clustered with HEV-1 and HEV-2 for ORF2 and clustered with HEV-2 for ORF3, but with low bootstrap support. Although different regions of the camel HEV genomes may be more similar to different genotypes of HEV, recombinational analysis performed using bootscan analysis revealed no obvious and definite site of recombination. Amino acid distances based on the concatenated ORF1/ORF2 excluding the HVR region of HEV-7 and HEV-8, and those of camel HEVs and the existing genotypes were ≥0.1, which was greater than the threshold (i.e., *p*-distance = 0.088) to demarcate inter-genotype distance [27] (Table 3), supporting that the camel HEVs should constitute two different HEV genotypes, for which HEV-7 and HEV-8 were proposed for DcHEV and BcHEV, respectively.

## 6. Delving into the Sequence: Recent Insights into HEV Molecular Epidemiology and Evolution 

Rapidly accumulating sequence information over the last two decades has enabled new insights into the evolution of HEV and its adaptation to new hosts. Such studies have largely focused on HEV-1, HEV-3 and HEV-4 in view of the relative importance to human health of these HEV genotypes and abundance of publicly available sequence information. Questions addressed include the ancient origins of HEV, reasons for current genotypic distribution, host restriction, zoonotic transmission, local epidemiological trends, and potential future evolutionary trends of this family. 

A recent systematic search for homologs of various HEV proteins in other viral families showed that the HEV1 RdRp protein sequence shares greater identity with insect viruses (*Alphatetraviridae*) and plant viruses (*Benyviridae*, *Betaflexiviridae* and *Virgaviridae*) compared to mammalian viruses [51]. However, phylogenetic analysis of the HEV ORF2 protein shows that it clusters together with members of the family *Astroviridae* and is quite distant from the aforementioned RdRp-homologous viral families; similarities in capsid crystal structure between astroviruses and HEV have also been noted lending credence to the theory of common ancestry [52,53]. Although convergent molecular evolution of genes encoding conserved enzymatic functions may theoretically be observed among unrelated viral families, this intriguing contrast in structural and non-structural protein phylogeny indicates a recombination event between the ORF1 sequence of insect/plant viruses and the ORF2 sequence of *Astroviridae* ancestors to produce a hybrid *Hepeviridae* ancestor. 

Divergence analysis of HEV1–4 suggests that the split into zoonotic and anthropotropic genotypes occurred approximately 536 to 1344 years ago although significant underestimation due to the limitations of evolutionary models is possible [54]. HEV-1 appears to be more recent than the zoonotic genotypes with the estimated time to most recent common ancestor (TMRCA) of most modern lineages of HEV-1 being ~87–199 years ago [54]. Population dynamics of HEV-1, HEV-3 and HEV-4 over the last century have also been described linking fluctuations in effective population size to global trade, wars, fluctuations in pork consumption, increased recognition of hepatitis E as a zoonosis and control measures in swine [54,55]. 

Comparison of non-synonymous (dN) and synonymous (dS) substitutions in each codon position of HEV genotypes 1, 3 and 4 reveals that most of the HEV genome is under purifying selection (dN < dS) irrespective of genotype. Exceptions are the hypervariable region of ORF1 and the ORF2/ORF3 overlap region, which appear to be under positive selection, a trend that is especially prominent in the zoonotic genotypes HEV-3 and HEV-4 [54,56]. Evolutionary rates differ between genotypes within *Orthohepevirus* A; HEV-3 and HEV-4 appear to have higher substitution rates over time and fewer conserved sites than HEV-1 across the entire genome [56]. Furthermore, codon usage bias of HEV-3 and HEV-4 ORFs tends to be lower than HEV-1 [57]. It is postulated that these findings may reflect the need of zoonotic genotypes to adapt to multiple hosts and therefore different cell types with distinct microenvironments and codon usage preferences. However, such host-specific adaptations at the molecular level could not be demonstrated in experimentally infected pigs inoculated with a HEV-3f isolate derived from a human [58]. Studies have attempted to link observed HEV sequence variations to viral host-specificity markers and inter-species transmissibility, but functional confirmation of such in silico analyses is still pending [59,60]. 

Evolutionary dynamics of HEV on a short-term local scale have been studied to explain the pattern of locally predominant subtypes. Transregional flow of HEV-4 across provinces in China has been demonstrated by phylogenetic and coalescent-based inference [61]. Similarly, Nakano et al. were able to demonstrate the indigenization of European HEV-3e variants in Mie prefecture of Japan in both humans and wild boar [62,63] probably through importation of large-race pigs from Europe approximately fifty years ago. Similar analyses have been conducted for HEV strains sequenced in Europe [64,65]. 

Intra-host viral evolution has also been recognized in immunocompromised individuals with chronic hepatitis E infection. Analogous to other chronic viral infections [66,67,68], HEV in chronically infected patients exists in the form of a cloud of closely-related variants which may potentially exhibit quasispecies behavior. These variants likely arise due to fluctuating immunological pressures from the host; composition of viral population tends to stabilize over time without treatment [69]. Such viral heterogeneity may have direct clinical consequences; Kamar et al. reported neurological symptoms in a renal transplant recipient with chronic HEV infection and identified distinct potentially neurotropic quasispecies in the patient’s cerebrospinal fluid [70], while Lhomme and colleagues have reported that increased quasispecies heterogeneity in the polyproline and macro regions of ORF1 may potentially be linked to chronic infections in immunocompromised hosts [71,72]. Viral heterogeneity is also of importance in patients receiving ribavirin treatment. While ribavirin may have multiple mechanisms of action including direct antiviral effects, depletion of intracellular guanosine triphosphate pools and immunomodulatory mechanisms, the prevailing hypothesis is that it induces viral mutagenesis at a rate beyond the error threshold pushing the population into extinction [73,74]. There is clear evidence for increased viral heterogeneity in ribavirin treated patients assessed using next generation sequencing. While ribavirin is the only effective antiviral available for chronic HEV infection, some patients are observed to be treatment refractory. In such patients, viral variants with mutations in the HEV RdRp region are selected out (e.g., G1634R, K1383N, D1384G, and K1398R) during treatment; the significance of these mutations in determining the ribavirin resistant phenotype is uncertain due to the difficulty in conducting in vitro studies and lack of widely accessible animal models [69,75,76]. 

While considerable advances have been made in the last decade towards an understanding of the molecular evolution and epidemiology of HEV variants of clinical importance, further high-quality complete genome sequences of other genotypes within *Orthohepevirus* A (including wild boar and camelid HEV) will be required so that these genotypes may be included in analyses. This is of vital importance due to the recent description of chronic camelid HEV infection in an immunocompromised patient [10] and possible transmission of wild boar HEV genotypes to humans [35], which indicates that zoonotic HEV from non-swine sources may be an emerging underestimated cause of hepatitis in humans. Further intensive studies to elucidate inter-species transmission patterns of these novel genotypes are required. 

## 7. Conclusions: Future Directions 

Our understanding of the diversity of the family *Hepeviridae* is constantly evolving. With accumulating sequence information and discovery of novel genotypes, it is likely that taxonomic classifications will require constant revisions to keep pace with advances. Future directions may include targeted discovery programs looking for novel *Orthohepevirus* A genotypes in artiodactyls living in close proximity with humans. Recent advances in metagenomic approaches and next-generation sequencing could greatly streamline this process and may potentially identify novel sources and routes of transmission. From the clinical perspective, due to the high degree of capsid structure amino acid identity among different genotypes within *Orthohepevirus* A, increasing evidence for zoonotic transmission from non-swine sources will be expected to accumulate. In particular, the importance of camelid hepatitis E genotypes in causing human hepatitis in enzootic areas urgently requires further study. Furthermore, constant vigilance for novel recombinants will be required. The ever-increasing cache of genomic data and sophisticated models will be useful to address questions related to HEV evolution and epidemiology; a preponderance of the extant data originates from Europe and East Asia, extending their approaches to other parts of the world will enable us to capture a global perspective and identify previously missed links. Finally, a better understanding of intra-host evolution of HEV during human infection may enable us to predict clinical outcomes and treatment response in patients at risk of chronic and fulminant HEV infection.

## Figures and Tables

**Table 1:** Comparison of nucleotide and deduced amino acid sequence identities of the most recently discovered genotype, HEV-8 (BcHEV-48XJ), and other genotypes of HEV *.

HEV Genotypes/Strains (GenBank Accession No.)	HEV-8 Strain BcHEV-48XJ (KX387866)						
	Nucleotide Identity (%)				Amino Acid Identity (%)		
	Entire Genome	ORF1	ORF2	ORF3	ORF1	ORF2	ORF3
*Orthohepevirus* A							
HEV-1							
Human HEV subtype 1a (M73218)	73.7	71.9	76.9	84.4	81.2	89.8	74.0
Human HEV subtype 1b (D11092)	73.8	71.8	77.2	84.1	81.1	90.0	74.0
Human HEV subtype 1b (L08816)	73.4	71.8	76.9	84.1	81.2	89.7	73.2
Human HEV subtype 1c (X98292)	73.7	71.6	77.4	84.1	81.5	89.5	75.6
Human HEV subtype 1d (AY230202)	74.3	72.1	78.4	84.7	81.6	90.2	74.0
Human HEV subtype 1e (AY204877) ^†^	73.4	71.6	77.6	84.4	79.8	90.2	74.0
Human HEV subtype 1f (JF443721)	73.8	71.7	77.8	79.9	81.0	90.3	80.7
HEV-2							
Human HEV subtype 2a (M74506) ^†^	73.2	72	76.1	84.1	80.4	88.3	74.0
HEV-3							
Mongoose HEV subtype 3a (AB591734)	75.8	74.2	78.5	85.9	85.7	93.0	71.3
Swine HEV subtype 3a (AF082843)	74.8	73.6	77.8	84.0	85.5	92.1	70.5
Wild deer HEV subtype 3b (AB189071)	75.3	73.9	78.5	84.8	85.6	92.6	71.3
Human HEV subtype 3b (AP003430)	75.3	73.6	78.3	85.6	85.9	92.9	73.0
Wild boar HEV subtype 3c (FJ705359)	75.8	74	78.8	85.4	86.4	92.6	72.1
Swine HEV subtype 3e (AB248521)	75.4	73.7	78.4	85.9	86.0	92.4	74.6
Human HEV subtype 3f (AB369687)	75.3	74	78.2	85.9	85.9	92.9	73.8
Human HEV subtype 3f (FJ653660)	75.5	74	78.2	85.4	85.9	92.4	73.8
Swine HEV subtype 3g (AF455784)	75.7	74.3	78.1	86.2	85.6	92.9	73.8
Human HEV subtype 3h (JQ013794) ^†^	74.6	73.1	78.1	79.1	85.3	92.6	78.8
Wild boar HEV subtype 3i (FJ998008) ^†^	75.1	73.9	78.0	84.0	86.0	92.9	70.5
Swine HEV subtype 3j (AY115488)	75.4	73.8	78.3	84.6	85.2	92.7	70.5
Rabbit HEV subtype 3ra (FJ906895)	74.1	71.7	78.4	82.4	82.2	88.6	70.5
HEV-4							
Human HEV subtype 4a (AB197673)	74.7	72.7	79.5	79.7	83.5	91.1	75.4
Swine HEV subtype 4b (DQ279091)	74.8	72.4	80.4	80.2	83.4	90.7	74.6
Human HEV subtype 4c (AB074915)	75.4	73.3	81.1	79.7	83.6	90.5	75.4
Human HEV subtype 4d (AJ272108)	75	73.2	79.3	77.5	83.6	88.9	70.2
Swine HEV subtype 4e (AY723745)	75.8	73.7	80.7	81.3	83.4	91.4	74.6
Human HEV subtype 4f (AB220974)	75.2	73.1	80.3	81.8	83.4	90.7	77.2
Human HEV subtype 4g (AB108537)	75.3	73.5	81.3	81.3	84.2	90.8	76.3
Swine HEV subtype 4h (GU119961)	75.2	73.1	79.9	79.9	82.7	90.1	76.3
Swine HEV subtype 4i (DQ450072)	75.5	73.2	80.0	87.0	82.7	92.4	73.0
HEV-5							
Wild boar HEV subtype 5a (AB573435)	74.4	72.4	79.4	75.3	82.1	87.8	72.6
HEV-6							
Wild boar HEV 6 (AB856243)	74.1	72.1	78.1	77.8	82.0	90.3	75.2
Wild boar HEV subtype 6a (AB602441)	74.5	73.2	76.5	77.5	81.3	89.8	73.7
HEV-7							
DcHEV 7 (KJ496144)	75.9	74	79.1	81.3	85.9	91.4	83.2
Human HEV 7 (KT818608) ^†^	73.4	74.5	69.2	NA	85.9	82.1	NA
DcHEV subtype 7a (KJ496143)	75.6	73.8	78.8	81.0	85.5	91.2	84.1
HEV-8							
BcHEV-62XJ (KX387867)	98.3	98.2	98.8	99.7	98.8	99.7	100.0
BcHEV-12XJ (KX387865)	96.3	96.1	97.0	97.8	98.0	99.2	99.1
*Orthohepevirus* B							
Avian HEV genotype 2 (AY535004)	52.9	52.8	51.6	42.3	43.2	44.8	24.8
*Orthohepevirus* C							
Germany rat HEV (GU345042)	57.1	56	59.9	54.2	49.8	55.1	27.4
Vietnam rat HEV (JX120573)	56.3	55.2	58.9	51.6	50.0	55.5	29.6
Ferret HEV (JN998606)	56.2	54.7	60.5	49.5	50.5	55.9	26.5
*Orthohepevirus* D							
Germany bat HEV (JQ001749)	53.5	53	54.8	47.0	43.7	47.3	22.9
*Piscihepevirus* A							
Cutthroat trout HEV (HQ731075)	48.1	48.5	47.9	37.4	28.4	20.2	15.5

* HEV, hepatitis E virus; BcHEV, HEV from Bactrian camel; DcHEV, HEV from dromedary camel; NA, not available because of incomplete genome. ^†^ Near-complete genome.

**Table 2:** Comparison of genomic organization of HEV genotypes and isolates *.

HEV Genotypes/Strains (GenBank Accession No.)	Genome Length, nt	GC Content, %	5′ UTR, nt	ORF1, aa	ORF2, aa	ORF3, aa	3′ UTR, nt
*Orthohepevirus* A							
HEV-1							
Human HEV1a (M73218)	7194	58.1	27	1693	660	114	65
Human HEV1b (D11092)	7194	57.7	27	1693	660	123	66
Human HEV1c (X98292)	7193	57.6	26	1693	660	123	65
Human HEV1d (AY230202)	7192	57.4	25	1693	660	123	65
Human HEV1e (AY204877) ^§^	≥7153	57.4	NA	≥1688	660	123	65
Human HEV1f (JF443721)	7194	57.7	27	1693	660	114	65
HEV-2							
Human HEV2a (M74506) ^‡^	≥7170	56.5	NA	1691	659	114	74
HEV-3							
Human HEV3a (AB089824)	7244	55.3	25	1709	660	113	72
Swine HEV3a (AF082843)	7207	55.6	9	1708	660	122	54
Human HEV3b (AP003430)	7230	55.3	26	1703	660	122	72
Wild boar HEV3c (FJ705359)	7222	55.5	25	1703	660	122	68
Swine HEV3e (AB248521)	7225	54.7	25	1704	660	122	68
Human HEV3f (AB369687)	7214	55.5	5	1704	660	122	77
Swine HEV3g (AF455784)	7215	55.9	28	1697	660	122	76
Human HEV3h (JQ013794) ^§^	≥7163	56.0	NA	≥1691	660	113	68
Wild boar HEV3i (FJ998008)	≥7197	55.6	NA	1703	660	122	68
Swine HEV3j (AY115488)	7242	55.4	26	1708	660	122	72
Rabbit HEV3ra (FJ906895) ^‡^	7283	55.5	26	1722	660	122	71
HEV-4							
Human HEV4a (AB197673)	7237	54.2	26	1706	674	114	69
Swine HEV4b (DQ279091)	7234	54.7	26	1705	674	114	69
Human HEV4c (AB074915)	7224	54.9	9	1707	674	114	70
Human HEV4d (AJ272108)	7232	54.4	25	1707	658	112	68
Swine HEV4e (AY723745)	7240	54.5	25	1707	674	114	70
Human HEV4f (AB220974)	7243	54.3	25	1707	674	114	73
Human HEV4g (AB108537)	7193	55.0	9	1706	674	114	42
Swine HEV4h (GU119961)	7240	55.1	26	1707	674	114	69
Swine HEV4i (DQ450072)	7235	55.2	26	1707	660	122	68
HEV-5							
Wild boar HEV5a (AB573435)	7250	55.5	25	1708	674	112	77
HEV-6							
Wild boar HEV6 (AB856243)	7247	55.6	25	1709	660	112	71
Wild boar HEV 6a (AB602441)	7246	57.0	25	1709	660	112	70
HEV-7							
Human HEV7 (KT818608) ^§^	7220	52.4	40	1698	660	113	66
DcHEV-178C (KJ496143) ^†^	7220	55.1	39	1698	660	113	66
DcHEV-180C (KJ496144) ^‡^	7219	54.4	39	1698	660	113	66
HEV-8							
BcHEV-12XJ (KX387865)	7223	52.7	26	1704	660	113	65
BcHEV-48XJ (KX387866)	7223	53.0	26	1704	660	113	65
BcHEV-62XJ (KX387867)	7212	53.1	15	1704	660	113	72
*Orthohepevirus* B							
Avian HEV genotype 1 (AM943647) ^§^	≥6627	55.1	NA	≥1531	606	87	123
Avian HEV genotype 2 (AY535004)	6654	55.5	24	1531	606	87	127
Avian HEV genotype 3 (AM943646)	≥6631	55.6	NA	1532	606	87	126
Avian HEV novel unclassified genotype (JN997392) ^§^	≥6543	55.7	NA	≥1515	606	87	NA
*Orthohepevirus* C							
Germany rat HEV (GU345042)	6948	57.8	10	1636	644	102	65
Vietnam rat HEV (JX120573)	6927	56.6	10	1629	644	102	65
Ferret HEV (JN998606)	6841	53.8	12	1596	654	108	65
*Orthohepevirus* D							
Bat HEV (JQ001749)	6767	51.8	33	1580	637	137	77
*Piscihepevirus* A							
Cutthroat trout HEV (HQ731075)	7269	49.7	100	1707	634	225	76

* HEV, hepatitis E virus; UTR, untranslated region; ORF, open reading frame; BcHEV, HEV from Bactrian camel; DcHEV, HEV from dromedary camel; NA, not available because of incomplete genome; ^†^ Assuming the third AUG of ORF2 is the start codon; ^‡^ Assuming the third AUG of ORF3 is the start codon; ^§^ Near-complete genome.

**Table 3:** *p*-Distance of the three BcHEV strains and other genotypes of HEV based on amino acid of ORF1/ORF2 excluding HVR region *.

HEV Genotypes/Strains (GenBank Accession No.)	*p*-Distance with Strain		
	BcHEV-12XJ	BcHEV-48XJ	BcHEV-62XJ
HEV-8			
BcHEV-12XJ	-	0.011	0.009
BcHEV-48XJ	0.011	-	0.006
BcHEV-62XJ	0.009	0.006	-
HEV-1			
Human HEV subtype 1a (M73218)	0.143	0.143	0.141
Human HEV subtype 1b (D11092)	0.141	0.142	0.140
Human HEV subtype 1c (X98292)	0.140	0.140	0.139
Human HEV subtype 1d (AY230202)	0.139	0.138	0.136
Human HEV subtype 1f (JF443721)	0.143	0.143	0.141
HEV-2			
Human HEV subtype 2a (M74506) ^†^	0.156	0.156	0.155
HEV-3			
Swine HEV subtype 3a (AF082843)	0.106	0.105	0.106
Human HEV subtype 3b (AP003430)	0.103	0.103	0.104
Wild boar HEV subtype 3c (FJ705359)	0.100	0.100	0.101
Swine HEV subtype 3e (AB248521)	0.104	0.105	0.105
Human HEV subtype 3f (AB369687)	0.105	0.105	0.105
Swine HEV subtype 3g (AF455784)	0.103	0.105	0.105
Wild boar HEV subtype 3i (FJ998008) ^†^	0.101	0.101	0.102
Swine HEV subtype 3j (AY115488)	0.109	0.109	0.110
Rabbit HEV subtype 3ra (FJ906895)	0.132	0.133	0.132
HEV-4			
Human HEV subtype 4a (AB197673)	0.122	0.121	0.120
Swine HEV subtype 4b (DQ279091)	0.124	0.121	0.120
Human HEV subtype 4c (AB074915)	0.123	0.121	0.120
Human HEV subtype 4d (AJ272108)	0.127	0.125	0.124
Swine HEV subtype 4e (AY723745)	0.120	0.119	0.119
Human HEV subtype 4f (AB220974)	0.127	0.125	0.124
Human HEV subtype 4g (AB108537)	0.121	0.119	0.118
Swine HEV subtype 4h (GU119961)	0.134	0.132	0.131
Swine HEV subtype 4i (DQ450072)	0.132	0.130	0.129
HEV-5			
Wild boar HEV subtype 5a (AB573435)	0.139	0.140	0.140
HEV-6			
Wild boar HEV 6 (AB856243)	0.139	0.140	0.139
Wild boar HEV subtype 6a (AB602441)	0.147	0.149	0.148
HEV-7			
DcHEV 7 (KJ496144)	0.108	0.107	0.106
Human HEV 7 (KT818608) ^†^	0.108	0.107	0.105
DcHEV subtype 7a (KJ496143)	0.110	0.109	0.108

* HEV, hepatitis E virus; BcHEV, HEV from Bactrian camel; DcHEV, HEV from dromedary camel; ORF, open reading frame; HVR, hypervariable region; ^†^ Near-complete genome.
